# Functional homogenization of flower visitor communities with urbanization

**DOI:** 10.1002/ece3.2009

**Published:** 2016-02-24

**Authors:** Nicolas Deguines, Romain Julliard, Mathieu de Flores, Colin Fontaine

**Affiliations:** ^1^ Department of Environmental Science, Policy, and ManagementUniversity of CaliforniaBerkeleyCalifornia; ^2^ MNHN‐CNRS‐UPMC UMR 7204 CESCOMuséum National d'Histoire NaturelleParisFrance; ^3^ Office Pour les Insectes et leur Environnement (OPIE)GuyancourtFrance

**Keywords:** Biotic homogenization, citizen science, flower visitor assemblages, macroecology, pollination, specialisation

## Abstract

Land‐use intensification and resulting habitat loss are put forward as the main causes of flower visitor decline. However, the impact of urbanization, the prime driver of land‐use intensification in Europe, is poorly studied. In particular, our understanding of whether and how it affects the composition and functioning of flower visitor assemblages is scant, yet required to cope with increasing urbanization worldwide. Here, we use a nation‐wide dataset of plant‐flower visitor (Coleoptera, Diptera, Hymenoptera, Lepidoptera) interactions sampled by citizen scientists following a standardized protocol to assess macroecological changes in richness and composition of flower visitor communities with urbanization. We measured the community composition by quantifying the relative occurrence of generalist and specialist flower visitors based on their specialisation on flowering plant families. We show that urbanization is associated with reduced flower visitor richness and a shift in community composition toward generalist insects, indicating a modification of the functional composition of communities. These results suggest that urbanization affects not only the richness of flower visitor assemblages but may also cause their large‐scale functional homogenization. Future research should focus on designing measures to reconcile urban development with flower visitor conservation.

## Introduction

Biodiversity decline continues (Butchart et al. 2010) with likely implications for the functioning of ecosystems (Cardinale et al. 2012). In particular, reports from several countries indicated that insect flower visitors, including many pollinators which provide both key ecosystem function and services (Dupont and Olesen 2009; Vanbergen and the Insect Pollinators Initiative 2013), experienced downward historical shifts [e.g. wild bees and hoverflies (Biesmeijer et al. 2006), and butterflies (Thomas et al. 2004)]. Habitat loss resulting from land‐use intensification was proposed as the main cause of flower visitor decline (Vanbergen and the Insect Pollinators Initiative 2013) as both the intensification of agricultural lands and practices (Kennedy et al. 2013) and urbanization (McKinney 2008; Bates et al. 2011) were found to decrease their richness.

Reports of changes in flower visitor diversity often rely on richness alone (Winfree et al. 2011). However, response to land‐use intensification can differ among species (Cariveau and Winfree 2015) as well as orders (Deguines et al. 2012; Verboven et al. 2014; Baldock et al. 2015), hence the need to measure compositional changes in flower visitor communities. For instance, Carvalheiro et al. (2013) found that the similarity of bee, hoverfly or butterfly assemblages increased between 1950 and 2009 in some European countries, revealing a taxonomic homogenization that decreases the overall diversity.

Previous works suggested that traits (e.g. voltinism, size, or specialisation regarding resource requirements) may be important in predicting which species are more prone to decline than others (Warren et al. 2001; Goulson et al. 2005; Biesmeijer et al. 2006). In particular, environmental changes are expected to be more detrimental to specialist species that have little success on the margins of their narrow ecological niche, compared to generalist species that can adapt more easily to varying environment (Clavel et al. 2010). Resulting changes in the functional composition of flower visitor communities may have important consequences for both pollination function (Fründ et al. 2013) and services (Hoehn et al. 2008). Whether land‐use intensification alters the functional composition of flower visitor communities is still poorly understood.

Here, we aim at deepening our understanding of the effects of the prime driver of land‐use intensification in Europe – urbanization (EEA 2010) – on flower visitor communities. Accounts on the natural history of anthophilous insects (Chinery 1986; Michener 2007) reveal how environmental changes associated with urbanization (e.g. increased impervious soil surfaces, reduced vegetation) can impact insect individuals and species (Cane 2005; Harrison and Winfree 2015). Assessing whether or not species‐level impacts of urbanization scale up to the community level is critical to the conservation of flower visitors and the ecosystem services they provide (Shwartz et al. 2013; Deguines et al. 2014; Potter and LeBuhn 2015). As urbanization reduces plant functional diversity (Knapp et al. 2008; Thompson and McCarthy 2008; Duncan et al. 2011), we expect a nonrandom loss of flower visitors according to their specialisation on floral resources that should result in changes in the functional composition of communities. Considering a broad taxonomic scope (Coleoptera, Diptera, Hymenoptera, and Lepidoptera) and a wide geographical scale (France), we assess spatial changes along an urbanization gradient in (1) flower visitor richness and (2) the relative composition of specialists and generalists using a continuous community‐level index of flower visitor specialisation on flowering plant families. We measure flower visitor community‐level changes along a spatial gradient of urbanization as a proxy for temporal variations associated with urbanization. We further analyze differences in flower visitor communities sampled on eight widely distributed plant families to inform on the generality of our findings and discuss implications for flower visitor conservation in urban areas.

## Material and Methods

### Dataset

**Figure 1:**
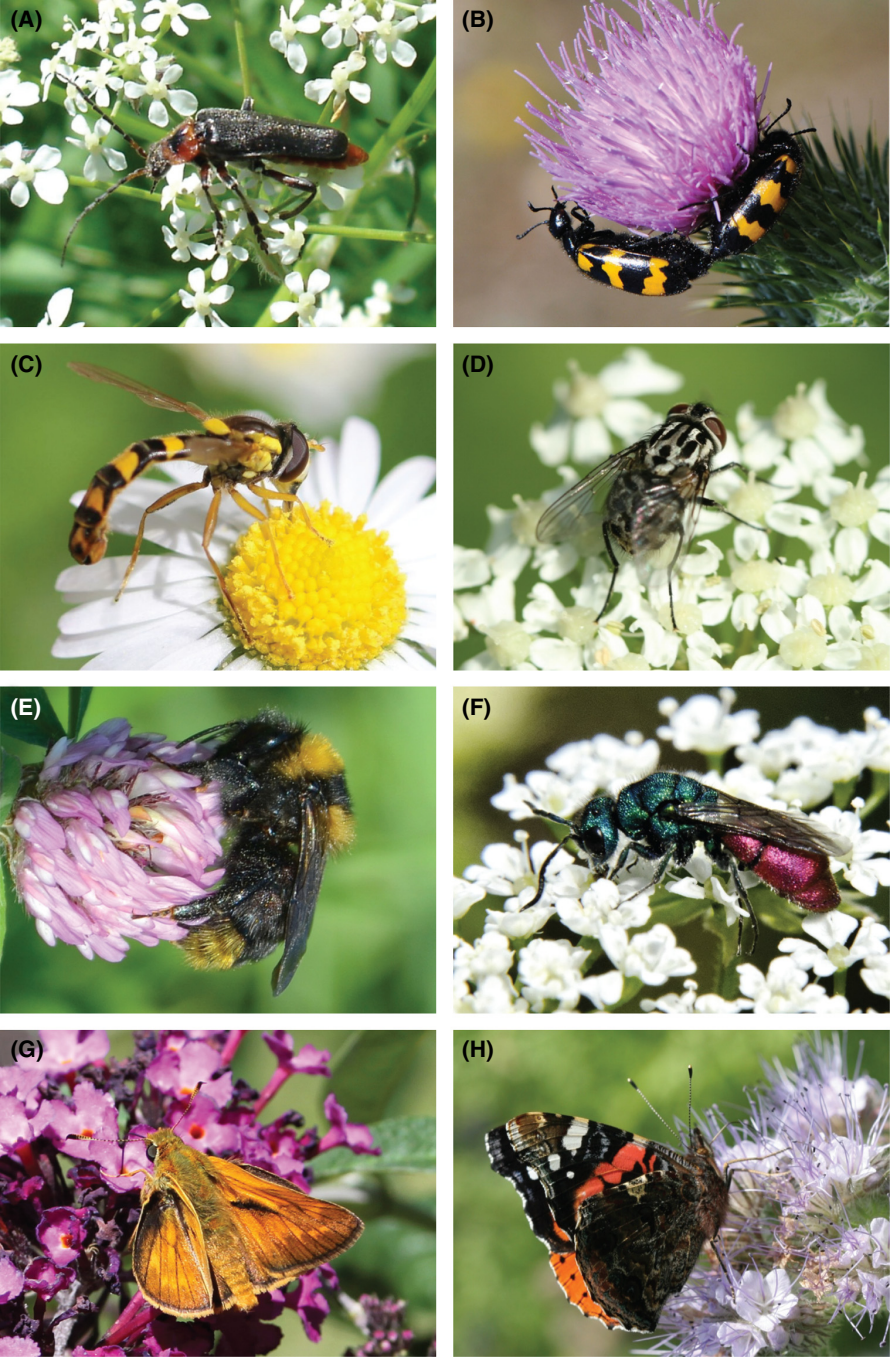
European pollinator communities are mostly composed of insects from the order Coleoptera (A, B), Diptera (C, D), Hymenoptera (E, F), and Lepidoptera (G, H). The Cantharids (A), the feather holder hoverfly (C), the yellow and black bumblebees (E) and the tawny skippers (G) are four instances of generalist pollinator taxa; the banded blister beetles (B), the Graphomya flies (D), the ruby tailed wasps (F) and the red admiral (H) are among the specialist pollinator taxa (see Table S1 for more details). Photographs credits: cvd – Spipoll (A), Prisca – Spipoll (B), cybelle – Spipoll (C), MichelMarly – Spipoll (D), calin01 – Spipoll (E), jfcth – Spipoll (F), Oxyna – Spipoll (G), Barbara Mai – Spipoll (H).

Flower visitors communities were sampled across France by participants of the Photographic Survey of Flower Visitors (hereafter Spipoll) who followed a standardized protocol fully described elsewhere (Deguines et al. 2012). Briefly, volunteers chose a flowering plant anywhere in France and took pictures of every insect visiting its flowers within a 20‐min period. Volunteers then named their plant and insects' pictures using online identification tools we developed and that provide a list of predefined taxa (i.e. morphospecies, defined as a group of species differing from all other groups in any external features consistently noticeable on pictures of plant flowers or free‐living arthropods). Identifications, first made by volunteers, are then validated by professional entomologists (see Acknowledgments). The 285 insect taxa on the list (Table S1) belong to the orders Coleoptera, Diptera, Hymenoptera, and Lepidoptera (Fig. 1). Their taxonomic resolution ranges from the family level (20 taxa) to species level (107 taxa) (Table S1). Date, time, temperature (<10°C, 10–20°C, 20–30°C, or >30°C), and precise location are provided by volunteers when uploading their data on the Spipoll's website (www.spipoll.org). Each set of plant and the insects that could be photographed visiting it is hereafter referred as a flower visitor collection.

We used data recorded by citizen scientists having contributed with a minimum of 20 flower visitor collections uploaded on the website to minimize differences among observers in the number of insects they photograph (see also Statistical analyses). We retained data collected under conditions allowing the observation of a whole range of anthophilous insects, that is between 8 am and 8 pm, at temperatures above 10°C, and from March to October.

We used QGIS (2015) to characterize the degree of urbanization surrounding flower visitor collections as the proportion of urban areas [“*Artificial surfaces*” in the first level of the Corine Land Cover 2006 database (Bossard et al. 2006)] in a 1‐km radius. Doing so, heterogeneous types of urban habitats (e.g. green spaces, residential areas, parking lot) are not differentiated as is the case in local studies investigating the determinants of flower visitor communities within urban areas (Hennig and Ghazoul 2012; Matteson et al. 2013). Our characterization thus does not allow examining the effects of the various aspects of urbanization but focuses instead on how flower visitor communities may change with the broad trend of increasing urbanization occurring at the detriment of agricultural or natural areas.

We used eight plant families (Apiaceae, Araliaceae, Asteraceae, Fabaceae, Lamiaceae, Malvaceae, Rosaceae, and Scrophulariaceae) to sample and measure the response of flower visitor collections to urbanization. These eight plant families were both largely sampled (range: 34–596, Table 1) and adequately distributed over the urbanization gradient, with a minimum of three records in each 0.2 increase in the proportion of urban areas. Additionally, these plant families encompassed contrasting flower morphologies and growth habits resulting in likely differential attractiveness to insects (Dupont and Olesen 2009). Thus, using several plant families should inform on the generality of potential changes in flower visitor communities along an urbanization gradient.

**Table 1:** Description of the dataset (1606 flower visitor collections) separated into two broad categories of urbanization degree

Nb. of samples	Proportion of urban areas (within 1 km)	
	Low ([0.0–0.50])	High ([0.5–1.0])
Total	1272	334
Per year	2010: 275, 2011: 511, 2012: 486.	2010: 147, 2011: 86, 2012: 101.
Per month	March: 78, April: 118, May: 173, June: 168, July: 282, August: 244, September: 147, October: 62.	March: 19, April: 19, May: 30, June: 38, July: 160, August: 32, September: 21, October: 15.
Per temperature category	10–20°C: 403, 20–30°C: 802, >30°C: 67.	10–20°C: 69, 20–30°C: 228, >30°C: 37.
Per plant family	Apiaceae: 250, Araliaceae: 35, Asteraceae: 484, Fabaceae: 128, Lamiaceae: 128, Malvaceae: 24, Rosaceae: 171, Scrophulariaceae: 52.	Apiaceae: 42, Araliaceae: 14, Asteraceae: 112, Fabaceae: 43, Lamiaceae: 50, Malvaceae: 10, Rosaceae: 37, Scrophulariaceae: 26.

“Nb. of samples” is the number of flower visitor collections.

**Figure 2:**
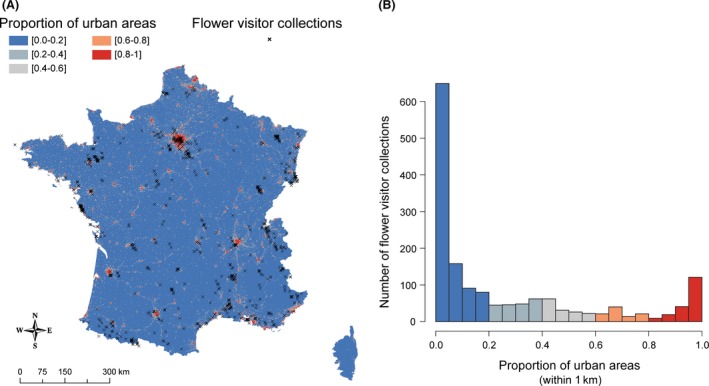
(A) The spatial distribution of the 1606 flower visitor collections (i.e. sampling sites) (black crosses) in France, with the proportion of urban areas within 1 km² squares increasing from blue to red. (B) A histogram of the proportion of urban areas in a 1‐km radius around the 1606 sample sites.

Following these filters, our dataset contained 7167 insects sampled on 1606 plants across France over 3 years (2010–2012) (Fig. 2).

### Flower visitor collections' indices

We characterized the richness and community‐level specialisation of flower visitors in each of the 1606 flower visitor collections (i.e. the set of flower visitors recorded visiting a given plant species in a 20‐min observation session).

We used the total number of taxa recorded in the collection as a proxy of flower visitor richness. Previous work validated the use of taxa richness measured by citizen scientists as an indicator of species richness (Kremen et al. 2011). The robustness of this indicator is further strengthened because, in this monitoring program, taxa are predefined in the identification tool. This maximizes consistency in taxa sorting across participants as taxa are not defined in various ways according to each volunteer's ability to detect insects' external differences, as can be the case in other methodologies relying on taxa (Obrist and Duelli 2010).

To obtain the Community Specialisation Index (CSI) of each of the 1606 flower visitor collections, we first characterized the specialisation of every insect taxon. To do so, we used the entire Spipoll dataset (3962 flower visitor collections sampled from 2010 to 2012) to calculate for every insect taxon that had a minimum of 20 records its specialisation toward the plant families it visited. This Specialisation Index (SI_i_) quantifies taxon i's departure from perfect generalism, defined as the visitation of plant families in proportion to their availability. We defined availabilities as the total number of samples of each plant family in the entire dataset (retaining only plant families with a minimum of 40 samples). The index is calculated following Julliard et al. (2006). The specialisation of taxon *i* toward plant family is calculated as follows:

$${\text{SI}}_{i}=\frac{\sqrt{\text{Var}(N_{i,f}/N_{f})}}{\text{Mean}(N_{i,f}/N_{f})}$$

where *N*_*i,f*_ represents the number of records of insect *i* on plants from family *f,* and *N*_*f*_ the total number of flower visitor collections sampled on plants from family *f*. The SI_i_ of an insect observed in exact proportion of the availability of plant families, that is a perfect generalist, would equal zero. Any shift in this distribution of insect visits, that is one or several family of plants are more or less visited than expected given their availability, is a specialisation that yields an increase in SI_i_. Finally, we obtained the CSI of each of the 1606 flower visitor collections by computing the average of SI_i_ across the insect taxa recorded in each collection. CSI increases with the relative occurrence of specialists within a collection. Insect taxa present in a community but with no defined SI_i_ (i.e. with less than 20 records in the entire dataset) were not included in the calculation of the CSI.

### Statistical analyses

All analyses were performed with the software R (R Core Team 2015). Flower visitor richness was included as the response variable in a generalized linear mixed‐effects model [R package *lme4*, (Bates et al. 2014)], using the Poisson family to model the error distribution. The proportion of urban areas surrounding flower visitor collections (continuous variable ranging from 0 to 1), the plant family (factor with 8 levels), as well as their interaction was included as fixed explanatory variables. We further included a set of explanatory variables which can influence flower visitor activity at different temporal scales of our dataset: the year of observation (factor with 3 levels, i.e. 2010, 2011 and 2012), the month of observation (coded as a continuous numerical variable ranging from 3 to 10, that is March–October, then scaled to achieve model convergence by dividing each value by 10) and its first order polynomial to account for seasonality in flower visitor activity, and the temperature in degree celsius (°C) (factor with 3 levels, i.e. 10–20°C, 20–30°C, and >30°C). We also adjusted the model for the geographical position of flower visitor collections by including their coordinates (longitude and latitude, standardized to achieve model convergence), their interactions and their first order polynomials to account for spatial autocorrelation (Legendre and Legendre 1998). We included the identity of the 60 observers as a random term on the intercept, to take into account that observers may differ in the number of insect they photograph.

To test whether the explanatory variables had an effect on the community specialisation index, we used a linear mixed‐effects model that included the same explanatory variables as described above. The error distribution was modeled with a Gaussian family, and we attributed prior weights (Bates et al. 2014) to flower visitor collections according to the proportion of insect that were available for the calculation of the CSI (i.e. for each collection, the proportion of insects with a defined specialisation index).

We used backward model simplification to obtain the minimum adequate structure of both models. Assumptions of homogeneity of variance and normality (for the CSI model) of the residuals were met. We found no evidence of spatial autocorrelation in the residuals of our models using both graphical assessment with spline correlograms (Zuur et al. 2009) and Moran's I index (richness model: *I *=* *2.3e^−5^, *P*‐value = 0.72; CSI model: *I *=* *−3.1e^−3^, *P*‐value = 0.17). We tested the effects of the explanatory variables with type‐III univariate analyses of variance (ANOVA). We further investigated differences among plant family with Tukey's honest significance tests and adjusting *P‐*values with the Bonferroni correction [R package *multcomp*, (Hothorn et al. 2014)]. We computed the conditional Pseudo‐R^2^ (R^2^_glmm_) of our models using the R package *MuMIn* (Barton 2014).

## Results

The 1606 sites of the flower visitor collections we analyzed in this study covered the whole France except Corsica (Fig. 2A), and the sites were distributed across the full urbanization gradient (Fig. 2B, Table 1). The mean number of insects recorded per flower visitor collection was 4.47 (95% confidence interval = 4.32–4.62%, range: 1–28), and the dataset contained 285 different insect taxa (Table S1), including 183 identified at least to the genus level (49% of the pictures) (Table 2).

**Table 2:** Number of insect taxa and observations (in brackets) recorded among orders and by taxonomic resolution

Taxonomic resolution	Coleoptera	Diptera	Hymenoptera	Lepidoptera
A whole family	7 (307)	7 (308)	3 (307)	3 (21)
Several genera within a family	6 (99)	5 (149)	5 (374)	4 (54)
Species from different genera	23 (492)	9 (758)	16 (532)	14 (272)
A genus	11 (85)	18 (466)	3 (50)	6 (39)
Species from a genus	7 (59)	2 (47)	16 (937)	13 (269)
A single species	24 (399)	25 (740)	11 (92)	47 (311)

### Flower visitor richness

**Figure 3:**
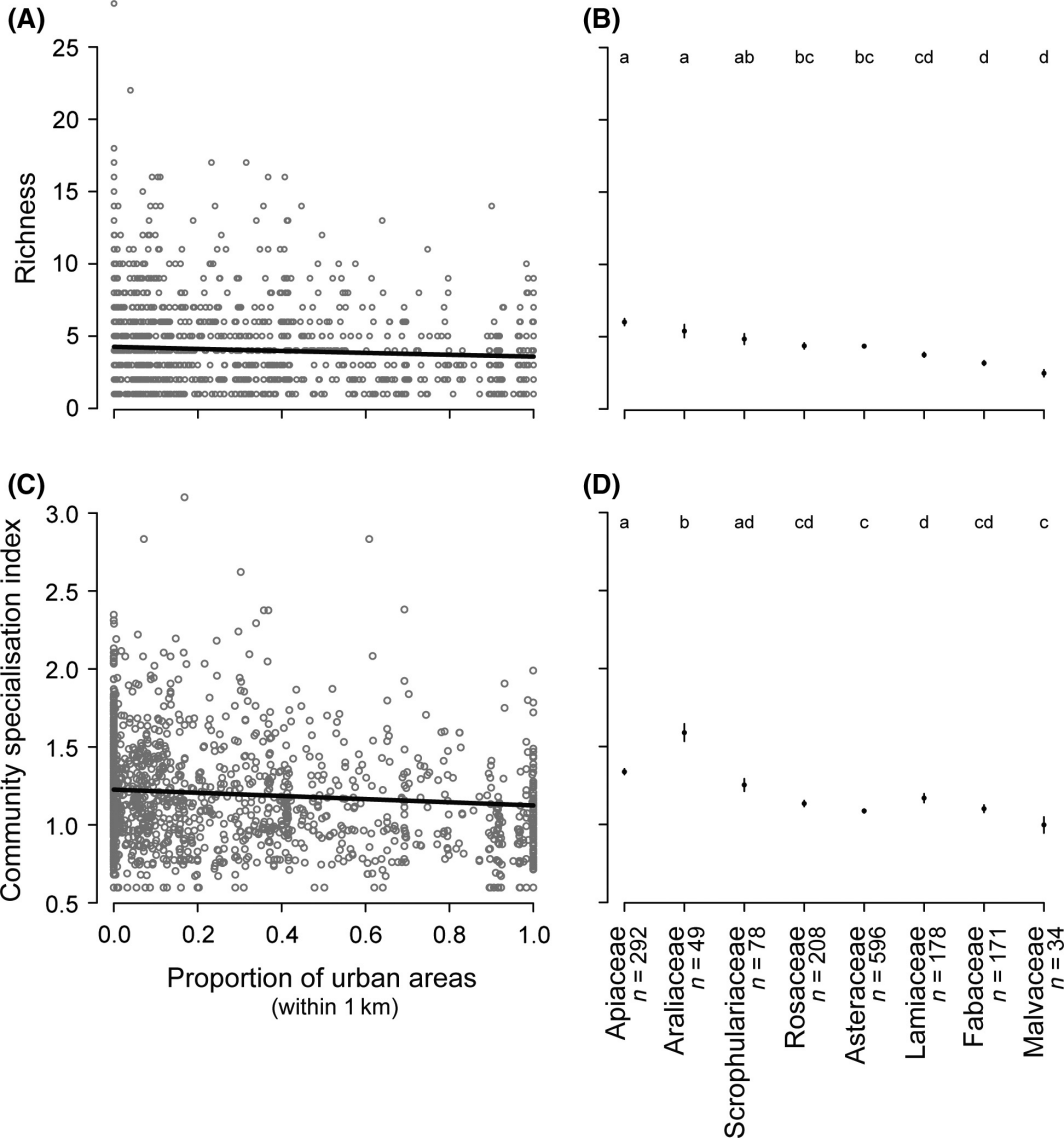
Variations in Richness (A, B) and the Community Specialisation Index (CSI) (C, D) according to the proportion of urban areas (within 1 km of sampling sites) (A, C) or the sampled plant family (B, D). In (A, C), gray circles are indicator values for each of the 1606 flower visitor collections and black curves represent the estimated trends retrieved from the models described in the methods. In (B, D), black dots and bars are mean values and associated standard errors for each plant family (with *n* the sampling size); letters indicate differences among plant families according to Tukey's honest significant tests (after accounting for multiple comparisons with the Bonferroni method). R^2^_glmm_ values for the richness and CSI model were 0.39 and 0.25, respectively.

We found a significant negative effect of the proportion of urban areas on flower visitor richness indicating that richness decreased with urbanization. This pattern was consistent across plant families as no significant interaction between the proportion of urban areas and plant family was found (Table 3, Fig. 3A). Additionally, plant family had a significant effect on flower visitor richness. Apiaceae and Araliaceae were visited by richer assemblages than Rosaceae, Asteraceae, Lamiaceae, Fabaceae, and Malvaceae. Scrophulariaceae, Rosaceae, and Asteraceae were themselves visited by richer insect community than Fabaceae and Malvaceae (Fig. 3B). The minimum adequate model's R^2^_glmm_ was 0.39, indicating that the set of remaining variables explained a substantial part of the variability in our data but also that unmeasured factors may be explanatory too.

**Table 3:** Type‐III ANOVA (*χ* ^2^ tests) results for the mixed‐effects models including Richness or CSI (Community Specialisation Index) as response variables. Degree of freedom (Df), *χ*² value, and *P*‐value are shown for the explanatory variables that remained in the minimum adequate models. “Urb”, “long”, and “lat” stand respectively for the proportion of urban areas in a 1‐km radius and the geographical position (standardized longitude, latitude) of flower visitor collections

Explanatory variables	Response variables					
	Richness			CSI		
	Df	*χ* ^2^ value	*P*‐value	Df	*χ* ^2^ value	*P*‐value
Urb	1	9.3923	0.002	1	10.746	0.001
Plant family	7	147.464	<0.001	7	218.861	<0.001
Year	2	57.720	<0.001	–	–	–
Month	1	64.261	<0.001	1	15.252	<0.001
Month^2^	1	64.041	<0.001	–	–	–
Temperature	2	9.176	0.010	–	–	–
Long	–	–	–	–	–	–
Lat	–	–	–	1	25.689	<0.001
long:lat	–	–	–	–	–	–
long^2^	–	–	–	–	–	–
lat^2^	–	–	–	–	–	–

Individually, the richness of orders Coleoptera, Diptera, and Lepidoptera decreased with increasing proportion of urban areas but this trend was only significant for Lepidoptera (Appendix S1). Hymenoptera richness nonsignificantly increased with urbanization.

### Community specialisation index

We found a significant negative effect of the proportion of urban areas on CSI, and this effect was independent of the plant family as shown by the absence of significant interaction between the proportion of urban areas and plant family (Table 3, Fig. 3C). This indicated that the relative occurrence of specialist and generalist taxa changed along the urbanization gradient, with flower visitor communities being more generalized in more urbanized areas. Additionally, there was a significant effect of plant family on CSI. Araliaceae attracted more specialist flower visitors than any of the other seven plant families. The CSI on Apiaceae was higher than on Rosaceae, Asteraceae, Lamiaceae, Fabaceae, and Malvaceae (Fig. 3D). The minimum adequate model explained a modest part of the variability in our data (R^2^_glmm_ = 0.25), suggesting that unmeasured factors may be important.

The CSI of each order tended to decrease with increasing proportion of urban areas although this trend was significant for Diptera only (Appendix S1).

## Discussion

We found that different facets of flower visitor diversity changed with urbanization, with not only a decreased richness but also an altered functional composition of flower visitor communities. While a review of the literature indicates that different taxonomic group may respond differently to urbanization (Bates et al. 2011; Deguines et al. 2012; Verboven et al. 2014; Baldock et al. 2015), we found that the richness of flower visitor communities as a whole decreased along the gradient of increasing urbanization. Separate analyses for each order suggested that Hymenoptera may not be as sensitive as the other three orders to urbanization (Appendix S1), in agreement with previous work (Deguines et al. 2012; Baldock et al. 2015). Most importantly, we found that urbanization is associated with changes in the composition of flower visitor communities which became biased toward generalist taxa. This result, along with the fact that each of the four orders followed a similar pattern (Appendix S1), is in agreement with our expectations and with previous work showing a similar trend for butterfly communities (Bergerot et al. 2011). Together with the decreasing richness observed with increasing proportion of urban areas in the landscape, this suggests that specialist flower visitors are lost during the urbanization process. Our findings therefore reveal the functional biotic homogenization of flower visitor communities along an urbanization gradient at a macroecological scale.

In this study, we estimated the specialisation of taxa with various levels of taxonomic resolution (from family to species, Table S1). Such variation in the taxonomic resolution could have an impact on the estimated level of specialisation, and thus on our results. For example, a taxon aggregating species from a given family could appear generalist whereas the species it is made of are all specialists. Such bias was indeed present in our data as there was a slight positive correlation between SI and taxonomic resolution (*Pearson's r *=* *0.171, *P*‐value = 0.062) indicating that less resolved taxa tend to be more generalists. However, our result indicating a decline in CSI associated with increased urbanization is robust against this bias because the mean taxonomic resolution of flower visitors is positively correlated with the proportion of urban areas (*r *=* *0.135, *P*‐value < 0.001), indicating that the taxonomic resolution of the insect taxa observed tend to be higher in urbanized area.

Patterns in urban sprawl may substantially vary between cities, and it therefore is difficult to generalize flower visitor response to urbanization from local studies (McKinney 2008). Our citizen science approach yielded a large dataset sampled in various local biophysical conditions (e.g. cities, plant family, temperature) and across 8 months for 3 years, thereby including a tremendous diversity of environmental conditions. Despite an important part of uncontrolled variability reflected by the modest amount of variations explained in our models (R^2^_glmm_ were 0.39 and 0.25 for the richness and CSI models, respectively), we were able to depict changes in flower visitor communities in concomitance with urbanization at a macroecological scale. Additionally, the similarity of effects across the eight plant families sampled suggests that the reduced richness and increased functional homogeneity associated with urbanization may be a general phenomenon. The high level of urbanization undergone since 1950s in Belgium, Great Britain, and the Netherlands (Fuchs et al. 2013) may then possibly explain the taxonomic homogenization of flower visitor assemblages observed during the past 60 years in these countries (Carvalheiro et al. 2013). At the rate at which urban areas increase at the continental scale [ca. 3% of net land‐use change between 2000 and 2006, i.e. 600 000 ha (EEA 2010)], urbanization therefore constitutes a major threat for flower visitor diversity in Europe.

The nested structure of plant–flower visitor interaction networks, where specialist species tend to interact with generalist species, increases plant tolerance to the loss of specialist flower visitors (Memmott et al. 2004). However, the foraging behavior of generalist species is modified in conditions of reduced interspecific competition such as less plant species are visited (Fründ et al. 2013). This results in the decreased ability of flower visitor communities being solely composed of generalists to provide a level of pollination function such as delivered by functionally diverse communities (Fründ et al. 2013). Therefore, following urbanization, the pollination function provided by resulting functionally homogenized flower visitor communities is likely to be altered, affecting the flora in consequence. Our findings thus suggest that urbanization could partially explain large‐scale decrease in insect‐pollinated plant richness associated with flower visitor decline (Biesmeijer et al. 2006).

Specialisation on flowering plant families appears to disadvantage flower visitors facing urbanization, most likely as a result of decreased plant functional diversity (Knapp et al. 2008; Thompson and McCarthy 2008; Duncan et al. 2011). Two plant families, Apiaceae and Araliaceae, attracted both richer and relatively more specialized communities than most other plant families. In particular, ivy species (Araliaceae) appeared particularly interesting for providing resources to specialist flower visitors (Fig. 3D), which confirms previous work underlining the value of these plants for flower visitor conservation in urban areas (Garbuzov and Ratnieks 2014). Nevertheless, the consistency of our results across families suggests that supporting specialist flower visitors in the colonization of urban areas would be more efficient through increasing plant functional diversity rather than sowing a single species. Establishing diverse urban wildflower meadows was found to drastically increase the abundance of bumble bees and hoverflies (Blackmore and Goulson 2014), and further work should assess their efficiency regarding the composition of flower visitor communities. The Scrophulariaceae, which mostly consisted in the exotic Butterfly bush (*Buddleja davidii,* 65% of the samples from this plant family), also attracted rich and specialized flower visitor community, in line with previous work suggesting that using both native and non‐native species may lead to an optimal management strategy (Salisbury et al. 2015). Such scheme however requires careful consideration because exotic species invading native ecosystems (as does the Butterfly bush) may have negative consequences (Ebeling et al. 2008).

Flower visitor traits are correlated with one another (Williams et al. 2010). In our study, the insect specialisation index was positively correlated with their phenologi‐cal specialisation (high specialisation corresponding to a narrow flight season) (*r *=* *0.299, *P*‐value < 0.001, Appendix S2), suggesting that long flowering period of urban plant flora could also benefit flower visitor communities. This is in agreement with a recent study suggesting that extending the season of resource provisioning to pollinators may benefit specialists in urban areas (Salisbury et al. 2015). Other studies identifying traits predicting flower visitor sensitivity to urbanization (e.g. nesting habits) are needed to better inform practitioners on management practices targeting flower visitors. This is a crucial step to strengthen already existing realistic compromises for embedding biodiversity in our cities (Snep et al. 2009), both for conservation objectives and the ecosystem services provided to urban citizens in need of nature (Shwartz et al. 2013) and crop pollination (McClintock 2010; Lowenstein et al. 2015; Potter and LeBuhn 2015).

## Conflict of Interest

None declared.

## Supporting information


**Appendix S1.** Separate analyses for the four flower visitor orders.

**Appendix S2.** Phenological specialisation.

**Table S1.** Description of the 285 flower visitor taxa.
