# Supplementary material for: Functional homogenization of flower visitor communities with urbanization
Source: Ecol Evol. 2016 Feb 24;6(7):1967–76. doi: 10.1002/ece3.2009 (PMC4767875; doi:10.1002/ece3.2009)
Supplement: Supplementary file 1 — Appendix S1. Separate analyses for the four flower visitor orders. [file ECE3-6-1967-s001.doc]

Appendix 1. Separate analyses for the four flower visitor orders

We present separate analyses for the four orders of flower visitors (Coleoptera, Diptera, Hymenoptera, Lepidoptera) as previous work have shown that each may respond differently to land-use changes (Bates et al. 2011, Deguines et al. 2012, Verboven et al. 2014, Baldock et al. 2015). The following analyses are based on smaller sample sizes (see Methods section below) and we thus invite readers to keep in mind this parameter while interpreting the results.

Methods

The four separate analyses follow the methods describe in the main text of our article. However, smaller datasets and partially different dataset are used because 1) no community specialisation index (CSI) can be calculated for a given order if no insect from that order is present in a flower visitor collection, and 2) we only analyse flower visitor collections from plant families with a minimum of three records in each 0.2 increase in the proportion of urban areas. Thus, resulting sample sizes for the separate analyses were 677, 843, 1159 and 337 for Coleoptera, Diptera, Hymenoptera and Lepidoptera respectively.

We used the same modelling approach as describe in the main text. All models met the assumptions of homogeneity of variance and normality (for the CSI models) of the residuals. We found no evidence of spatial autocorrelation in the residuals of our models, neither from spline correlograms nor from Moran’s I index (Coleoptera richness model’s *I*= -0.003, *p-value*=0.78, and CSI model’s *I*= -4.3e-4, *p-value*=0.80; Diptera richness model’s *I*= -0.006, *p-value*=0.19, and CSI model’s *I*= -0.004, *p-value*=0.31; Hymenoptera richness model’s *I*= 0.003, *p-value*=0.16, and CSI model’s *I*= -0.004, *p-value*=0.19; Lepidoptera richness model’s *I*= 0.007, *p-value*=0.27, and CSI model’s *I*= -0.012, *p-value*=0.28).

Results and discussion

*Richness*

We found a significant negative effect of the proportion of urban areas on the richness of Lepidoptera (Fig. A1G) (Table A1). The richness of Coleoptera and Diptera tended to decrease with increasing proportion of urban areas but the effect was not significant (Fig. A1A, C). Conversely, the richness of Hymenoptera tended to increase with increasing proportion of urban areas but this effect was not significant (Fig. A1E). These trends are mostly in agreement with the results presented in the main text although Hymenoptera may be more tolerant to urbanisation, as suggested in previous studies (Deguines et al. 2012, Baldock et al. 2015).

*Community specialisation index*

We found a significant negative effect of the proportion of urban areas on the community specialisation index of Diptera (Fig. A1D) (Table A1). The community specialisation index of the remaining three orders tended to decrease with increasing proportion of urban areas but effects were not significant (Fig. A1B, F, H). These trends are in agreement with the results presented in the main text and suggest that all four orders may suffer from a functional biotic homogenization associated with urbanisation.

Figure A1. Variations in Richness (A, C, E, G) and the Community Specialisation Index (B, D, F, H) according to the proportion of urban areas (within 1km of sampling sites) for Coleoptera (A, B), Diptera (C, D), Hymenoptera (E, F) and Lepidoptera (G, H). Grey circles are indicator values for each of the 677, 843, 1159 and 337 Coleoptera, Diptera, Hymenoptera and Lepidoptera collections. Black curves (solid = significant, dashed = non-significant) represent the estimated trends retrieved from the models described in the methods of Appendix 2, with their estimate and associated p value provided on top of each panel. When there was no significant effect of the variable “proportion of urban areas”, we used the last model that included this variable before it was removed during the backward model simplification procedure to retrieve the estimate of the trend (Table A1).


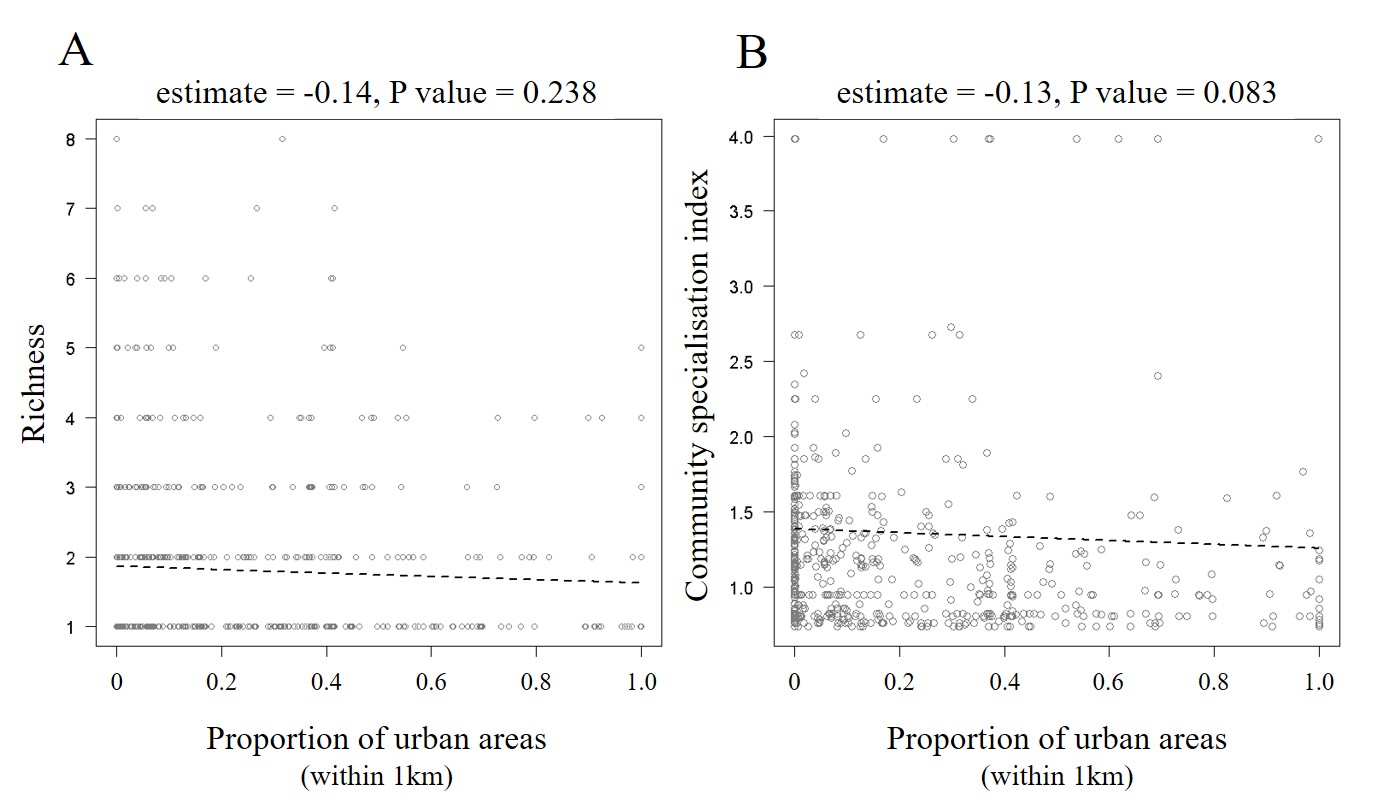


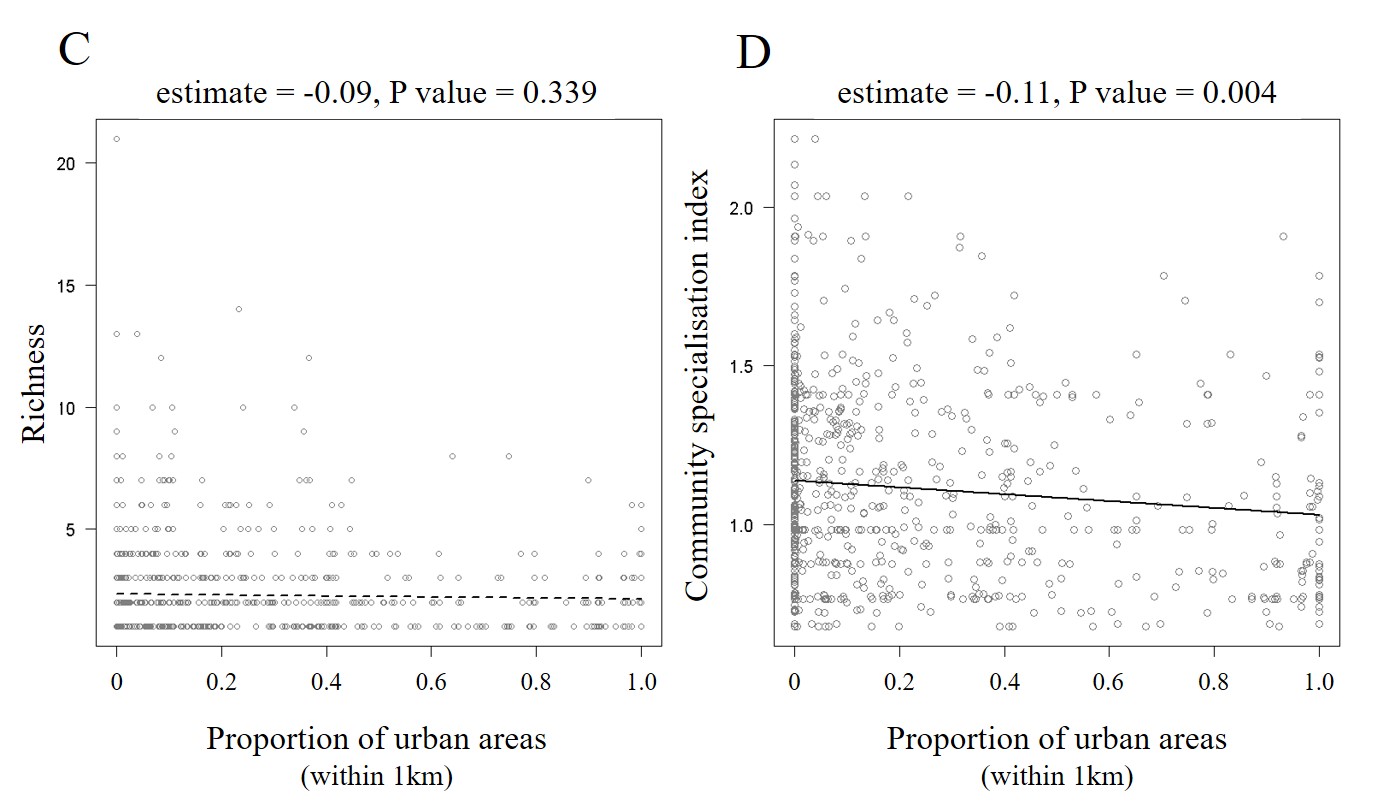


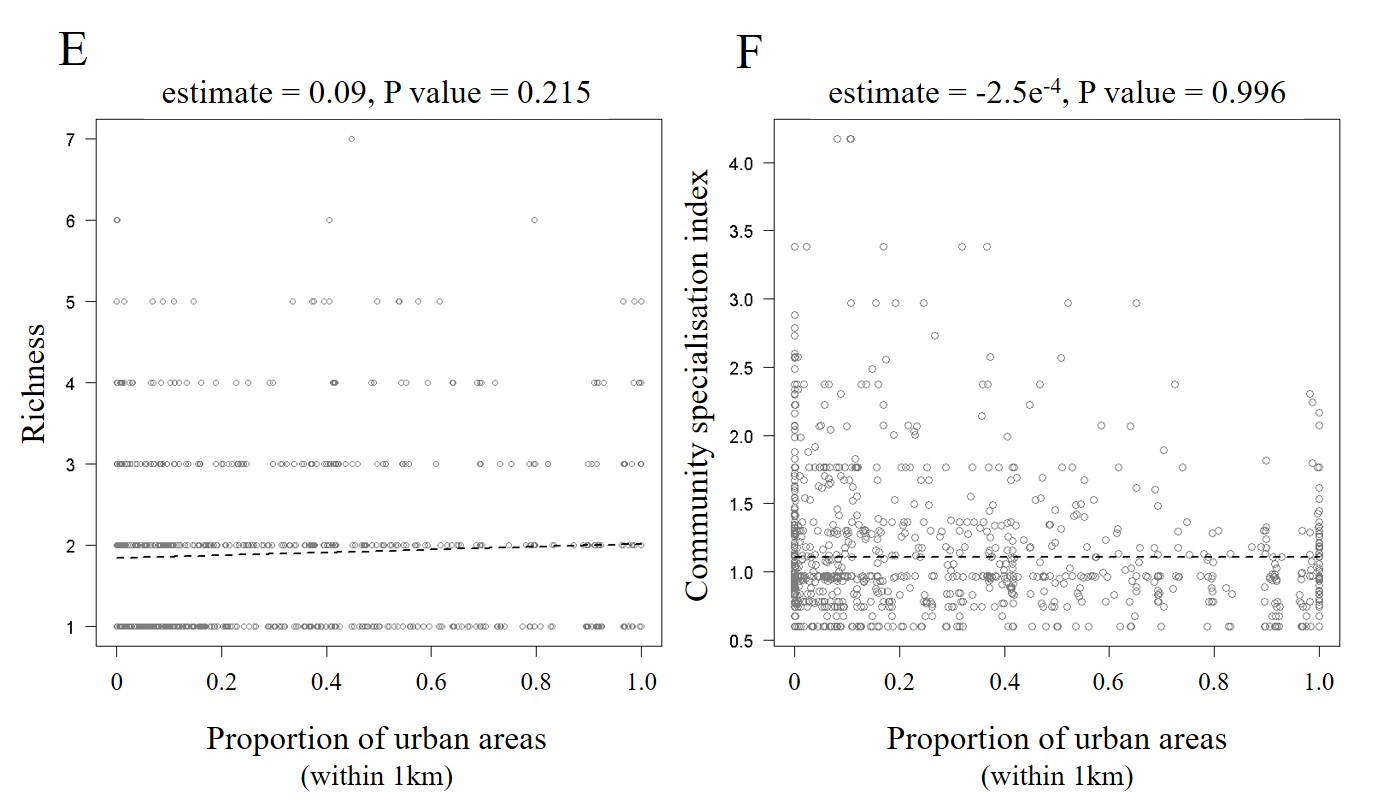


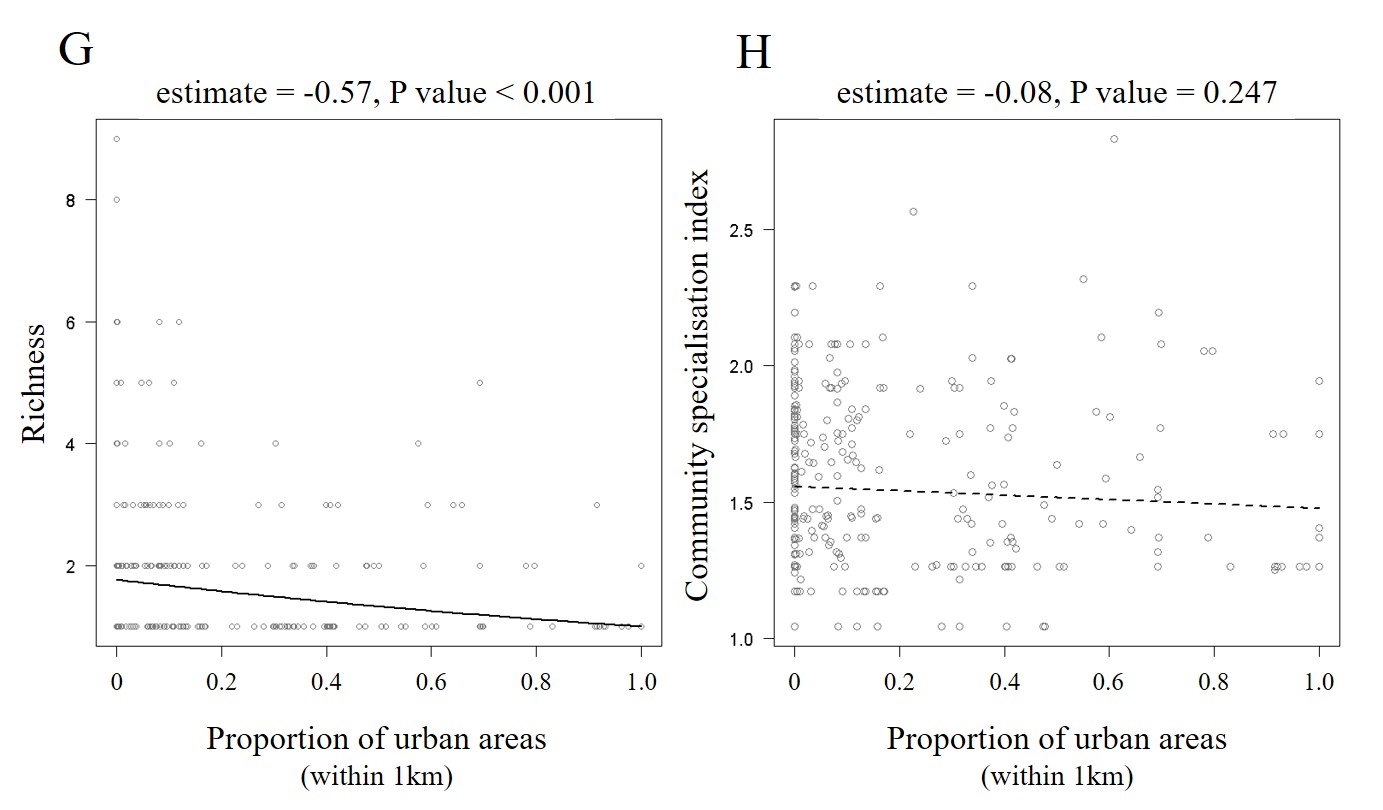


Table A1. Type-III ANOVA (χ² tests) results for the mixed-effects models including Richness or CSI (Community Specialisation Index) of each of the four orders as response variables. Degree of freedom (Df), χ² value, and P value are shown for the explanatory variables that remained in the minimum adequate models. ‘Urb’, ‘long’ and ‘lat’ stand respectively for the proportion of urban areas in a 1-km radius and the geographical position (standardized longitude, latitude) of flower visitor collections.

|  |  |  |  |  |  |  |  |
| --- | --- | --- | --- | --- | --- | --- | --- |
| **Explanatory variables** | **Response variables** | | | | | | |
|  | **Coleoptera** | | | | | | |
|  | **Richness** | | |  | **CSI** | | |
|  | Df | χ² value | P value |  | Df | χ² value | P value |
| Urb | 1 | 1.394 | 0.238 |  | 1 | 3.002 | 0.083 |
| Plant family | 4 | 34.403 | <0.001*** |  | 4 | 99.897 | <0.001*** |
| Year | - | - | - |  | - | - | - |
| Month | 1 | 39.407 | <0.001*** |  | 1 | 4.875 | 0.027 |
| Month² | 1 | 47.790 | <0.001*** |  | 1 | 4.129 | 0.042 |
| Temperature | - | - | - |  | 2 | 5.438 | 0.066 |
| long | - | - | - |  | - | - | - |
| lat | - | - | - |  | 1 | 25.689 | <0.001*** |
| long:lat | - | - | - |  | - | - | - |
| long² | 1 | 1.5322 | 0.216 |  | - | - | - |
| lat² | - | - | - |  | - | - | - |
|  |  |  |  |  |  |  |  |
|  | **Diptera** | | | | | | |
|  | **Richness** | | |  | **CSI** | | |
|  | Df | χ² value | P value |  | Df | χ² value | P value |
| Urb | 1 | 0.913 | 0.339 |  | 1 | 8.194 | 0.004** |
| Plant family | 4 | 80.445 | <0.001*** |  | 4 | 3.736 | <0.001*** |
| Year | 2 | 21.487 | <0.001*** |  | - | - | - |
| Month | 1 | 2.991 | 0.084 |  | 1 | 10.504 | 0.001** |
| Month² | 1 | 1.941 | 0.164 |  | - | - | - |
| Temperature | 2 | 2.927 | 0.231 |  | - | - | - |
| long | 1 | 11.155 | <0.001*** |  | 1 | 4.587 | 0.032* |
| lat | 1 | 4.143 | 0.042* |  | - | - | - |
| long:lat | - | - | - |  | - | - | - |
| long² | 1 | 3.437 | 0.064 |  | - | - | - |
| lat² | - | - | - |  | - | - | - |
|  | **Hymenoptera** | | | | | | |
|  | **Richness** | | |  | **CSI** | | |
|  | Df | χ² value | P value |  | Df | χ² value | P value |
| Urb | 1 | 1.534 | 0.215 |  | 1 | 0.000 | 0.996 |
| Plant family | 6 | 18.467 | 0.005** |  | 6 | 477.387 | <0.001*** |
| Year | - | - | - |  | 2 | 3.352 | 0.187 |
| Month | - | - | - |  | 1 | 1.450 | 0.228 |
| Month² | - | - | - |  | 1 | 1.210 | 0.271 |
| Temperature | 2 | 7.094 | 0.029* |  | 2 | 0.414 | 0.813 |
| long | - | - | - |  | 1 | 2.145 | 0.143 |
| lat | - | - | - |  | 1 | 1.131 | 0.288 |
| long:lat | - | - | - |  | 1 | 0.202 | 0.653 |
| long² | - | - | - |  | 1 | 3.648 | 0.056 |
| lat² | - | - | - |  | 1 | 0.499 | 0.480 |
|  | **Lepidoptera** | | | | | | |
|  | **Richness** | | |  | **CSI** | | |
|  | Df | χ² value | P value |  | Df | χ² value | P value |
| Urb | 1 | 11.028 | <0.001*** |  | 1 | 1.338 | 0.247 |
| Plant family | - | - | - |  | 2 | 3.736 | 0.154 |
| Year | - | - | - |  | 1 | 1.846 | 0.174 |
| Month | - | - | - |  | 1 | 2.016 | 0.156 |
| Month² | - | - | - |  | - | - | - |
| Temperature | 2 | 12.181 | <0.001*** |  | - | - | - |
| long | - | - | - |  | - | - | - |
| lat | - | - | - |  | 1 | 7.311 | 0.007 |
| long:lat | - | - | - |  | - | - | - |
| long² | - | - | - |  | - | - | - |
| lat² | - | - | - |  | - | - | - |

Literature Cited

Baldock, K. C. R., M. A. Goddard, D. M. Hicks, W. E. Kunin, N. Mitschunas, L. M. Osgathorpe, S. G. Potts, K. M. Robertson, A. V. Scott, G. N. Stone, I. P. Vaughan, and J. Memmott. 2015. Where is the UK’s pollinator biodiversity? The importance of urban areas for flower-visiting insects. Proceedings of the Royal Society of London B: Biological Sciences 282:20142849.

Bates, A. J., J. P. Sadler, A. J. Fairbrass, S. J. Falk, J. D. Hale, and T. J. Matthews. 2011. Changing bee and hoverfly pollinator assemblages along an urban-rural gradient. PLoS ONE 6:e23459.

Deguines, N., R. Julliard, M. de Flores, and C. Fontaine. 2012. The whereabouts of flower visitors: contrasting land-use preferences revealed by a country-wide survey based on citizen science. PLoS ONE 7:e45822.

Verboven, H. A. F., R. Uyttenbroeck, R. Brys, and M. Hermy. 2014. Different responses of bees and hoverflies to land use in an urban–rural gradient show the importance of the nature of the rural land use. Landscape and Urban Planning 126:31–41.
