# Supplementary material for: Functional homogenization of flower visitor communities with urbanization
Source: Ecol Evol. 2016 Feb 24;6(7):1967–76. doi: 10.1002/ece3.2009 (PMC4767875; doi:10.1002/ece3.2009)
Supplement: Supplementary file 2 — Appendix S2. Phenological specialisation. [file ECE3-6-1967-s002.doc]

Appendix 2: Phenological specialisation

In this study, we focused on flower visitor feeding specialisation at the level of plant family (SI). This trait is both a response trait (it may drive a species response to environmental changes, in this case urbanization) and an effect trait (it determines to a certain extent the role of a species in the ecosystem) (Lavorel and Garnier 2002), the latter with direct implications for the functioning of plant-flower visitor networks. However, flower visitor traits may be correlated with each other (see Table 2 in (Williams et al. 2010)). We built a Phenological Specialisation Index (PSI) and investigated its potential correlation with the Specialisation Index (SI).

Methods

For each insect taxon *i*, we calculated a phenological specialisation index (PSI_i_). Investigating the temporal distribution of records from taxon *i* on the eight months from March to October, its calculation is as follows:

where *N_i,m_* represents the number of records of insect *i* in month *m* and *N_m_* the total number of flower visitor collections sampled in month *m*.

We then performed a test for the correlation between SI and PSI using Pearson’s product-moment correlation coefficient using R (R Core Team 2013).

Results

There was a slight but significant positive correlation between SI and PSI (*Pearson’s r* = 0.299, *p-value* < 0.001, Fig. A1). This indicates that flower visitors showing specialisation on the families of plant they visit tend to be phenologically specialized, i.e. to have a narrow flight season.

Figure A1. Variations in the phenological specialisation index (PSI) with the specialisation index (SI). Each grey circle is one of the 120 flower visitor taxa for which specialisation indexes were calculated (i.e. with 20 records or more) (Table S1).


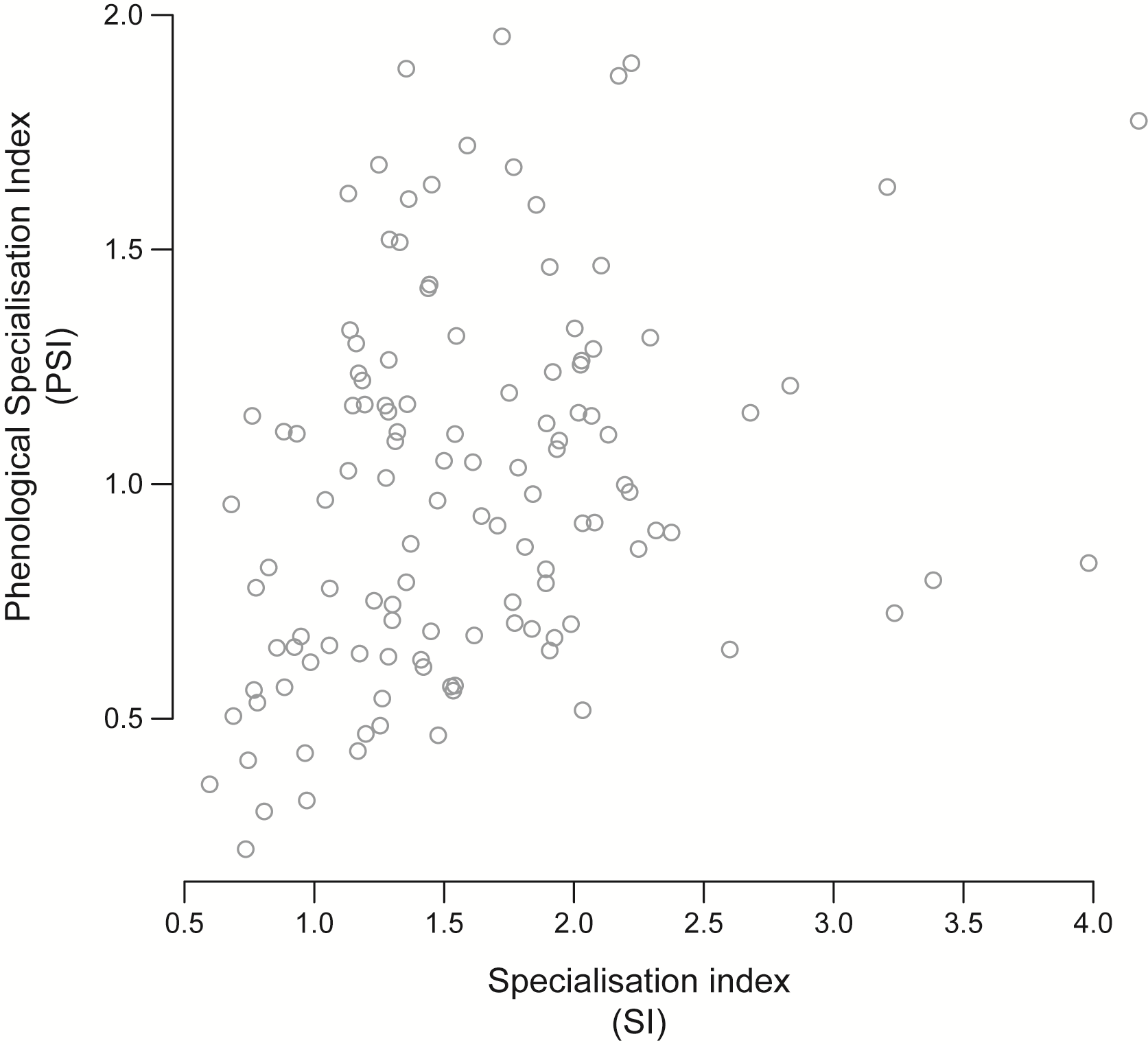


Literature Cited

Lavorel, S., and E. Garnier. 2002. Predicting changes in community composition and ecosystem functioning from plant traits: revisiting the Holy Grail. Functional Ecology 16:545–556.

R Core Team. 2013. R: a language and environment for statistical computing. R Foundation for Statistical Computing, Vienna, Austria.

Williams, N. M., E. E. Crone, T. H. Roulston, R. L. Minckley, L. Packer, and S. G. Potts. 2010. Ecological and life-history traits predict bee species responses to environmental disturbances. Biological Conservation 143:2280–2291.
